# Benefits of hypocrisy: Do managers gain more from greenwashing

**DOI:** 10.1371/journal.pone.0353235

**Published:** 2026-07-10

**Authors:** Weixin Dong, Youcai Yang, Yan Chen

**Affiliations:** 1 College of Economics and Management, Qingdao University of Science and Technology, Qingdao, Shandong, China; 2 College of Economics and Management, Qingdao University of Science and Technology, Qingdao, Shandong, China; 3 The Center for Economic Research, Shandong University, Jinan, Shandong, China; University of Durham: Durham University, UNITED KINGDOM OF GREAT BRITAIN AND NORTHERN IRELAND

## Abstract

This study finds that corporate greenwashing raises executive compensation. Mechanism analysis indicates that greenwashing enhances executive compensation by improving short-term performance and enhancing executive reputation. In addition, this effect is more pronounced in non-state-owned enterprises, companies with equity incentive plans, and firms that grant restricted stock.

## Introduction

Greenwashing refers to the practice of companies disseminating inadequate or misleading environmentally friendly claims to appease regulators and the public, thereby enhancing their reputations, even when their genuine green efforts are limited [1]. Extant studies have demonstrated various detrimental effects of greenwashing, such as reducing long-term stock returns [2], decreasing labor investment efficiency [3], and diminishing corporate market value [4]. Given these negative impacts of greenwashing, why do companies still engage in it? What motivates persistent greenwashing, and who, ultimately, benefits from it? This study shifts the analytical lens from the firm to the individual executive, guiding our investigation with the following core research question: Do corporate executives personally gain from greenwashing through increased compensation?

Current studies have identified various factors influencing executive compensation, including executive competence [5], risk appetites [6], and the market environment [7]. The managerial entrenchment theory posits that managers may interfere with the quality of accounting information to protect their positions and private interests [8]. Under weak internal and external scrutiny, managers may disclose misleading sustainable information through greenwashing, thereby earning revenue in the short term [9]. Research has shown that short-term performance improvement, even if achieved at the expense of long-term corporate development, can elevate executive compensation [10].

We propose two mechanisms through which greenwashing influences executive compensation. First, rooted in principal-agent theory, executive compensation is frequently tied to short-term performance targets. Improved short-term performance is often interpreted as a positive signal of executive capability. This perception, in turn, can lead to increased compensation. Greenwashing can generate short-term economic benefits, such as obtaining low-cost financing and boosting sales [11]. These gains are subsequently reflected in financial statements as apparent performance improvements. Second, the executive reputation channel functions through the personal reputational gains that executives derive from greenwashing. By associating themselves with prominent environmental initiatives, executives cultivate an image as visionary and socially responsible leaders. This enhanced image bolsters their managerial reputations and increases their bargaining power during compensation negotiations [12]. Therefore, we posit that greenwashing serves as a strategic tool that enables executives to improve both short-term company performance and their personal reputations, ultimately leading to higher compensation.

Furthermore, we propose that this relationship is not uniform across all firms but is contingent on specific organizational contexts. First, non-state-owned enterprises typically face stronger market competition and have more flexible compensation structures linked to short-term outcomes compared to state-owned enterprises, which are subject to stricter regulatory oversight and different incentive schemes. We thus conjecture that the positive effect of greenwashing on executive compensation will be more substantial in non-state-owned enterprises. Second, the presence of equity-based incentives amplifies executives’ motivation to engage in and benefit from greenwashing. In firms where a significant portion of executive compensation is tied to stock performance or other equity-related metrics, managers have a stronger impetus to boost short-term market perceptions and financial indicators. Greenwashing, by creating a favorable corporate image and potentially elevating stock valuations, enables executives to directly capitalize on these equity-linked rewards. Thus, we hypothesize that the effect of greenwashing on compensation is more pronounced in firms that implement equity incentive schemes. Finally, the effects of equity incentives vary by their type. Stock options are designed to encourage long-term stock price growth, while restricted stock awards are typically contingent on achieving short-term performance targets. Consequently, in firms that adopt restricted stock as a compensation scheme, the effect of greenwashing on compensation is more pronounced.

Our study makes two contributions to the literature. First, it deepens the understanding of greenwashing’s economic consequences. While most existing research focuses on the determinants of greenwashing [3], we systematically reveal its significant economic implications from an executive compensation perspective. More importantly, we find that while greenwashing creates potential risks for firms, it simultaneously generates private benefits for top managers at the personal level. This evidence provides a crucial explanation for the persistent prevalence of greenwashing despite its documented negative consequences. Second, this research expands the boundaries of the determinants of executive compensation. Traditional studies have primarily concentrated on factors such as corporate financial performance, market size, and industry characteristics [5]. By introducing a greenwashing perspective, we reveal how executives may enhance their personal compensation through symbolic environmental promotion rather than substantive environmental improvements. Consequently, we propose linking executive compensation to verifiable environmental performance and penalizing greenwashing behavior, thereby shifting incentives from symbolic communication to substantive commitment and addressing this issue at its root cause.

## Literature review

### The consequences of greenwashing

Greenwashing refers to corporate practices of disseminating misleading environmental information and exaggerating environmental protection efforts [13–15]. Existing literature has extensively documented the negative impacts of corporate greenwashing.

The prevalence of greenwashing has increased significantly alongside rising corporate environmental performance claims [16], creating substantial noise in information environments that impedes external investors’ ability to assess firm value accurately. This information asymmetry can trigger fluctuations in investor sentiment, leading to stock mispricing and ultimately impairing capital market efficiency [17,18]. From the consumer perspective, the intentional dissemination of misleading green information erodes trust in corporate reputation and product quality. Consequently, stakeholders may become reluctant to invest, and consumers may be less willing to purchase such products [19,20]. When greenwashing practices are exposed, firms face not only reputational damage but also regulatory penalties and additional sanctions [21,22]. At the corporate level, although greenwashing may yield short-term profits, its long-term benefits are often negligible and, in some cases, may even result in significant losses. Once discovered, organizations face regulatory penalties and reputational damage, leading to financial losses [23]. From the market valuation standpoint, greenwashing can trigger negative assessments in capital markets, adversely affecting financial performance and ultimately diminishing firm value [24,25]. Moreover, when certain companies temporarily benefit from greenwashing, others may replicate these practices, creating a ripple effect that spreads across industries or regions and ultimately harms overall societal welfare [26].

Despite these predominantly negative characterizations, emerging evidence suggests that greenwashing may offer short-term advantages, such as enhanced corporate image or immediate financial gains, which could potentially benefit individual actors within the firm [27]. However, the literature has largely overlooked how these symbolic practices might translate into personal gains for executives, setting the stage for exploring intersections with executive compensation research.

### Determinants of executive compensation

The determinants of executive compensation are widely regarded as a central topic in corporate governance literature. Traditional perspectives, rooted in principal-agent theory, advocate closely linking executive pay to firm performance to align managerial interests with those of shareholders, thereby forming the theoretical foundation for compensation design [28,29]. However, a growing body of empirical evidence indicates that the factors influencing executive compensation extend well beyond financial performance. These non-performance factors can be broadly categorized into two groups: managerial characteristics and power, and external market and institutional environments.

First, managers’ personal traits and the organizational power structure significantly influence their compensation. Grounded in managerial power theory, CEOs with greater influence can secure excess compensation through bargaining with boards of directors [30,31]. Furthermore, the “personal capital” accumulated through executives’ backgrounds and behaviors serves as valuable bargaining chips in compensation negotiations. Specifically, executives or directors with political connections typically receive higher compensation [32,33]. Kang and Kim (2017) [34] demonstrate that CEOs with greater media exposure and positive public images request higher pay, reflecting returns from reputation markets. Executives possessing generalist skills [35] or foreign experiences [36] also receive compensation premiums due to their scarcity and comprehensive capabilities. Additionally, CEOs’ risk aversion directly influences compensation structure, with risk-averse executives preferring higher fixed pay over performance-based incentives [37].

Second, firm-level characteristics and external institutional environments substantially impact compensation determination. Firm size is among the most robust factors, as larger firms offer higher compensation due to their complexity and need to attract qualified executives [38,39]. Policy environments also play a crucial role, as evidenced by the significant share of tax incentives that translate into executive compensation, demonstrating how institutional gains are allocated at the executive level [40].

While existing studies on executive compensation have comprehensively examined tangible performance metrics, personal traits, and institutional factors, they largely overlooked the influence of symbolic corporate actions, such as environmental disclosures, on pay. For instance, greenwashing behaviors have been documented in the literature for their potential short-term benefits to corporate reputation and performance. This raises the question of whether such benefits motivate executives to strategically exploit greenwashing to enhance their personal economic interests. Accordingly, our study bridges these research gaps by proposing that greenwashing serves as a mechanism through which executives pursue private gains, ultimately resulting in higher compensation.

## Data, variables, and method

### Data sources and model specifications

Data includes all listed companies in China from 2010 to 2022. The greenwashing indicator is measured from the corporation’s annual report. Executive compensation and other financial indicators are obtained from the China Stock Market & Accounting Research (CSMAR) database. We exclude financial firms, samples with abnormal trading status (ST, *ST), and observations with missing values. All continuous variables are winsorized at the 1% and 99% percentiles. Eventually, we obtain 12,312 firm-year observations.

#### Dependent variable: Executive compensation (*Mpay*).

Following Murphy and Sandino (2020) [41], the dependent variable of executives’ compensation is measured by the average compensation of executives (directors, supervisors, and senior executives).

#### Explanatory variable: Greenwashing (*GWS*).

The core theoretical concept of greenwashing refers to the disparity between corporate environmental claims and actual performance. Following the approach proposed by Zhang et al. (2022) [42], greenwashing is quantified by calculating the difference between standardized ESG disclosure scores and standardized ESG actual performance. Compared to single-dimensional ESG ratings, this measurement strategy effectively distinguishes symbolic communication from substantive actions, providing a basis for identifying greenwashing in corporate environmental strategies. Specifically, we employ the Bloomberg ESG disclosure score to assess ESG disclosure (*ESG_dis*). This metric primarily relies on objectively disclosed information—including annual reports, statutory filings, sustainability reports, and corporate websites—and emphasizes quantitative breadth over qualitative depth, thus serving as a transparent indicator of reporting extensiveness without capturing substantive performance [43]. In contrast, we use the Hua Zheng ESG disclosure score to gauge ESG actual performance (*ESG_real*) [Data source is: https://www.chindices.com/esg-ratings.html.]. This metric’s evaluation framework incorporates China’s institutional and market characteristics, focusing not only on policy commitments but also emphasizing the implementation and tangible outcomes of ESG activities [44]. The quantitative formula of the greenwashing variable is expressed in equation (1):


$$\begin{array}{c} \begin{array}{c} {GWS}_{it}=\left|\left(\frac{{ESG}_{dis,it}-\overset{―}{{ESG}_{dis}}}{\sigma_{dis}}\right)-\left(\frac{{ESG}_{real,it}-\overset{―}{{ESG}_{real}}}{\sigma_{real}}\right)\right| \end{array} \end{array}$$ (1)

where $\overset{―}{{ESG}_{dis}}$, $\overset{―}{{ESG}_{real}}$, $\sigma_{dis}$, and $\sigma_{real}$ represent the mean and standard deviation of two ESG measures, respectively. ${GWS}_{it}$ represents the degree of greenwashing of company *i* in year *t*. The higher the value, the higher the degree of greenwashing.

#### Control variables.

While our study focuses on the link between greenwashing and executive compensation, we recognize the influence of other factors, including firm and executive characteristics, as well as corporate governance structures. Referring to Hu et al. (2022) [29], Di and Li (2023) [3], and Zhang et al. (2025) [45], control variables used in our study can be categorized as:

(1) Company characteristics: (i) company size (*Size*), represented as the natural logarithm of total assets; (ii) firm age (*Firmage*), determined as the natural logarithm of the number of years since the firm’s establishment; (iii) leverage ratio (*Lev*), evaluated as total liabilities divided by total assets; (iv) operating income growth rate (*Growth*), measured as increased operating income for the year over operating income for the previous year;
(2) Executive characteristics: (v) age of executives (*Gage*), indicated as the average age of corporate executives; (vi) gender ratio(*Gender*), measured as the proportion of male executives; (vii) length of service (*Tenure*), calculated as average number of years executives have been in their current tenure; (viii) CEO duality indicator (*Dual*), indicated as a dummy variable equal to 1 if the chairperson also serves as CEO and 0 otherwise;
(3) Corporate governance characteristics: (ix) institutional investor holdings (*Inst*), measured by the proportion of total shares held by institutional investors relative to the total number of shares outstanding; (x) independent directors (*Inep*), calculation of the proportion of independent directors among the total number of directors; (xi) and shareholder holdings (*Top5*), measured as the shareholding ratios of the top five shareholders.

#### Model specification.

Lastly, we construct a panel data regression model to examine the relationship between greenwashing and executive compensations:


$$\begin{array}{c} \begin{array}{c} {MPay}_{it}=\alpha_{\text{0}}+\alpha_{\text{1}}{GWS}_{it}+\alpha_{\text{2}}{control}_{it}+\partial_{i}+\theta_{t}+\varepsilon_{it} \end{array} \end{array}$$ (2)

We also controlled for relevant variables that may influence executive compensation levels. In addition, $\partial_{i}$ and $\theta_{t}$ are firm- and year-fixed effects, respectively, and $\varepsilon_{it}$ is the random disturbance.

### Summary statistics

Table 1 reports the summary statistics. The mean value of our primary dependent variable, greenwashing, is −0.012, with a standard deviation of 1.178. These results indicate the presence of, and wide variation in greenwashing behaviors among different firms. The mean value of the executive compensation is 15.623, with a standard deviation of 0.770. Its maximum and minimum values are 17.694 and 13.842, respectively, indicating significant variations in executive compensation across different listed firms. Firm size and age have average values of 23.207 and 2.919, respectively. Additionally, the leverage ratio and operating income growth rate are about 0.480 and 0.178, respectively. Male executives account for approximately 83.1% of corporate leadership positions. The average age and tenure of corporate executives are 50.17 and 3.89 years, respectively. The average values for the CEO duality indicator and the ratio of independent directors are approximately 21.2% and 37.6%. Lastly, the investor’s shareholding ratio and the average shareholding ratio of the top five shareholders are 50.2% and 55.4%, respectively.

**Table 1:** Summary statistics.

VarName	Obs	Mean	SD	Min	Median	Max
*GWS*	12,347	−0.012	1.178	−2.701	−0.053	3.409
*Mpay*	12,347	15.623	0.770	13.842	15.587	17.694
*Size*	12,347	23.207	1.323	20.523	23.092	26.961
*Firmage*	12,347	2.919	0.343	1.792	2.996	3.526
*Lev*	12,347	0.480	0.200	0.068	0.491	0.888
*Growth*	12,347	0.178	0.368	−0.493	0.119	2.177
*Gender*	12,347	0.831	0.105	0.539	0.846	1.000
*Gage*	12,347	50.165	3.080	42.470	50.210	57.380
*Tenure*	12,347	3.887	1.512	0.983	3.703	7.905
*Dual*	12,347	0.212	0.409	0.000	0.000	1.000
*Inst*	12,347	0.502	0.220	0.023	0.521	0.915
*Indep*	12,347	0.376	0.055	0.333	0.364	0.571
*Top5*	12,347	0.554	0.165	0.194	0.552	0.912

Table 2 presents the correlation matrix for the core variables. A significant positive correlation is observed between greenwashing and executive compensation, providing preliminary support for our main hypothesis. Furthermore, most control variables show statistically significant relationships with executive compensation, except for management gender and the proportion of independent directors. These preliminary findings warrant further investigation through multivariate regression analysis to rigorously test our research hypotheses.

**Table 2:** Pairwise correlation coefficients.

	*Mpay*	*GWS*	*Size*	*Firmage*	*Lev*	*Growth*	*Gender*
*Mpay*	1						
*GWS*	0.088***	1					
*Size*	0.465***	0.078***	1				
*Firmage*	0.170***	−0.022**	0.173***	1			
*Lev*	0.106***	−0.056***	0.499***	0.140***	1		
*Growth*	0.039***	0.058***	−0.004	−0.100***	−0.003	1	
*Gender*	−0.014	−0.006	0.185***	−0.090***	0.160***	−0.039***	1
*Gage*	0.159***	0.017*	0.400***	0.198***	0.127***	−0.131***	0.265***
*Tenure*	0.136***	−0.074***	−0.00400	0.100***	−0.068***	−0.071***	−0.040***
*Dual*	0.044***	0.050***	−0.097***	−0.055***	−0.097***	0.063***	−0.132***
*Inst*	0.138***	0.046***	0.366***	0.067***	0.141***	−0.033***	0.136***
*Indep*	−0.008	0.028***	0.081***	−0.039***	0.014	−0.002	−0.046***
*Top5*	0.026***	0.109***	0.265***	−0.200***	0.037***	0.050***	0.107***
	*gage*	*tenure*	*dual*	*inst*	*indep*	*top5*	
*Gage*	1						
*Tenure*	0.207***	1					
*Dual*	−0.150***	0.039***	1				
*Inst*	0.267***	0.009	−0.139***	1			
*Indep*	0.059***	0.006	0.078***	0.028***	1		
*Top5*	0.142***	−0.204***	−0.040***	0.509***	0.069***	1	

Note: *, **, and *** indicate significance levels of 0.10, 0.05, and 0.01, respectively.

## Empirical results

### Main results

Table 3 sequentially incorporates control variables for firm characteristics, executive characteristics, and corporate governance characteristics. In Columns (1) and (4), whether or not control variables are added, the estimated coefficients of *GWS* are positive and significant at the 1% level. This suggests that greenwashing has a significant impact on increasing executive compensation levels.

**Table 3:** Main results.

	(1)	(2)	(3)	(4)
	*MPay*	*MPay*	*MPay*	*MPay*
*GWS*	0.012***	0.011***	0.011***	0.011***
	(2.825)	(2.714)	(2.746)	(2.806)
*Size*		0.338***	0.337***	0.336***
		(30.414)	(29.739)	(29.696)
*Firmage*		−0.071	−0.102	−0.121*
		(−1.047)	(−1.490)	(−1.743)
*Lev*		−0.342***	−0.347***	−0.357***
		(−7.716)	(−7.847)	(−8.059)
*Growth*		0.011	0.013	0.015
		(1.022)	(1.198)	(1.339)
*Gender*			−0.016	−0.012
			(−0.237)	(−0.179)
*Gage*			−0.009***	−0.008***
			(−3.148)	(−3.010)
*Tenure*			0.018***	0.018***
			(4.743)	(4.671)
*Dual*			−0.039***	−0.034**
			(−2.886)	(−2.518)
*Inst*				−0.003
				(−0.117)
*Indep*				−0.792***
				(−7.533)
*Top5*				−0.078
				(−1.222)
cons	15.623***	8.145***	8.666***	9.070***
	(4792.046)	(26.594)	(25.860)	(26.378)
*N*	12,312	12,312	12,312	12,312
adj. *R*^2^	0.779	0.805	0.806	0.807
*Year FE*	Yes	Yes	Yes	Yes
*Firm FE*	Yes	Yes	Yes	Yes

Note: t-statistics are reported in parentheses; *, **, and *** indicate significance levels of 0.10, 0.05, and 0.01, respectively. All specifications include firm and year fixed effects and use heteroskedasticity-robust standard errors.

### Robustness tests

We perform robustness tests by: (1) We use the natural logarithm of the total compensation of the top three executives (*RMPay*) as an alternative measure to reassess executive compensation. (2) Following Hu et al. (2023) [46], we define greenwashing as the discrepancy between disclosed and actual environmental performance (*GWSS*). (3) We adopt the methodology of Wang et al. (2024) [47] to calculate ESG rating differences (*ESG rating*). This paper ranks ESG ratings from six providers—*Bloomberg, Wind, SynTao Green Finance, Menglang, Huazheng, and FTSE Russell*—and uses the overall mean adjusted for pairwise standard deviations as the measurement metric [ESG data on *Wind, SynTao Green Financ, and FTSE Russell* are obtained from the Wind Financial Terminal (https://www.wind.com.cn/). Data source of *Menglang* is available at https://www.susallwave.com/]. (4) We adjust standard errors at both the firm and year levels by employing the two-way clustering method. (5) We incorporate multiple high-dimensional fixed effects, including province- and industry-specific trend fixed effects. Results in Columns (1) to (6) of Table 4 confirm that our main findings remain robust.

**Table 4:** Robustness tests.

	(1) Alternative measures	(2) Alternative measures	(3) Alternative measures	(4) Adjust clustering	(5) High-dimensional fixed	(6) High-dimensional fixed
	*RMPay*	*MPay*	*MPay*	*MPay*	*MPay*	*MPay*
*GWS*	0.014***			0.011***	0.011***	0.011***
	(3.794)			(2.806)	(2.752)	(2.784)
*GWSS*		0.029**				
		(2.091)				
*ESG rating*			0.082***			
			(4.610)			
control	Yes	Yes	Yes	Yes	Yes	Yes
cons	8.643***	9.092***	9.161***	9.070***	9.373***	9.589***
	(25.602)	(26.436)	(26.635)	(26.378)	(27.080)	(27.831)
N	12,312	12,312	12,312	12,312	12,312	12,312
adj. R^2^	0.819	0.807	0.808	0.807	0.808	0.807
Year FE	Yes	Yes	Yes	Yes	Yes	Yes
Firm FE	Yes	Yes	Yes	Yes	Yes	Yes
Clustering (Firm*Year)	No	No	No	Yes	No	No
Prov_Year FE	No	No	No	No	Yes	No
Ind_Year FE	No	No	No	No	No	Yes

Note: t-statistics are reported in parentheses; *, **, and *** indicate significance levels of 0.10, 0.05, and 0.01, respectively. All specifications include firm and year fixed effects and use heteroskedasticity-robust standard errors.

### Endogeneity tests

Addressing endogeneity is crucial when examining the impact of greenwashing on executive compensation. The primary endogeneity concerns in this study arise from omitted variable bias and reverse causality. First, despite controlling for a range of firm and executive characteristics, unobservable factors simultaneously influencing both a company’s greenwashing propensity and executive compensation levels may still exist. For instance, hard-to-quantify variables such as a company’s internal governance culture or an executive’s personal risk-taking propensity may both drive management toward more speculative greenwashing strategies and directly influence their bargaining power in compensation negotiations. Ignoring these variables could bias the coefficient estimates for the core explanatory variables. Second, our study also faces questions regarding reverse causality. Executives who already command high salaries and greater power may have stronger motivation and capacity to manipulate corporate image to consolidate their position and justify their value, thereby engaging in greenwashing more frequently [48].

To alleviate the aforementioned concerns regarding endogeneity, we conduct endogeneity tests using the following methods:

(1) We adopt the Propensity Score Matching (PSM) method to mitigate endogeneity issues arising from selection bias. Following recent research [49,50], we use a 1:3 nearest-neighbor matching setting to conduct our analysis. According to Rassen et al. (2012) [51], this setting can balance statistical power and matching precision. Rassen et al. (2012) [51] claim that when the control group sample size is relatively large, 1:3 matching can preserve a larger sample size without substantially increasing bias, thereby offering higher statistical efficiency than 1:1 matching. Furthermore, we conduct a robustness check using 1:1 nearest-neighbor matching, which confirms the stability of our main findings.
(2) Our study employs Oster’s sensitivity analysis to alleviate omitted variable bias [52]. First, we estimate the bias-corrected statistic, denoted as β*. If the interval between the estimated coefficient *β* and the β* statistic does not include zero, it indicates that the baseline estimate is not solely influenced by unobserved variables. Second, the *δ* statistic is estimated. If the estimates for *δ* exceed 1, it suggests that the baseline results are not significantly affected by the absence of confounding variables.
(3) We adopt the System-GMM method to estimate a dynamic panel-data regression model by incorporating the lagged *GWS* as an additional explanatory variable. This approach uses levels and differences of lagged variables of *GWS* as instruments to address potential endogeneity. To ensure the validity of these instruments, we perform the Hansen test of over-identifying restrictions. We expect a p-value greater than 0.10 to fail to reject the null, indicating that the instruments are valid. In our results, the Hansen p-value is 0.205, which is above these thresholds and confirms instrument validity. Additionally, we assess the presence of autocorrelation in the first-differenced errors. These tests check the conditions for instrument validity in dynamic panel models. Specifically, due to the first-differencing process, we expect first-order serial correlation (AR(1)) to be present and statistically significant. However, second-order serial correlation (AR(2)) should be absent and insignificant; its presence would indicate that the lagged instruments are correlated with the errors, invalidating them. In our estimation, the AR(1) coefficient is −15.45 and significant (p < 0.01), while the AR (2) coefficient is 0.14 and insignificant, satisfying these conditions and supporting the model’s validity.
(4) Lastly, we use the instrumental variables method to address endogeneity issues. Specifically, we use the industry-region average level of *GWS* as an instrumental variable. This instrument is theoretically relevant because firms in the same industry and region face similar environmental pressures and norms, leading to correlation with the firm’s own GWS, but it is exogenous because the average GWS of peers should not directly affect the focal firm’s executive compensation except through its own GWS. In Table 5, Column (5), the Kleibergen-Paap rk LM statistic tests for potential underidentification. A low p-value rejects this null, indicating that the model is identified. Our p-value of 0.000 strongly rejects the null, confirming identification. Furthermore, the Kleibergen-Paap rk Wald F statistic is a robust test for weak instruments. It assesses whether the instruments are strongly correlated with the endogenous variable. Values greater than 16.38 (a common rule-of-thumb based on Stock-Yogo critical values for 10% maximal IV bias) indicate strong instruments, reducing concerns about weak instrument bias. Our statistic of 2397.454 far exceeds this threshold, confirming that the instrument is reliable.

**Table 5:** Endogeneity tests.

	(1) PSM 1:3	(2) PSM 1:1	(3) Oster’s sensitivity analysis		(4) GMM	(5) IV
	*MPay*	*MPay*	Bounds	Delta	*MPay*	*MPay*
*GWS*	0.010^**^	0.009^*^	[0.005, 0.020]	2.971	0.014*	0.027**
	(2.570)	(1.917)			(1.928)	(2.193)
Excluded zero			Yes			
δ>1				Yes		
control	Yes	Yes	Yes	Yes	Yes	Yes
AR (1)					−15.45***	
AR (2)					0.14	
Hansen-p					56.88 [0.205]	
Kleibergen–Paap rk LM Test						1159.414*** (0.000)
Kleibergen-Paap rk Wald F statistic						2397.454 [16.38]
*N*	11,693	9,483	--	--	10,791	12,304
Firm FE	0.808	Yes	Yes	Yes	Yes	Yes
Year FE	Yes	Yes	Yes	Yes	Yes	Yes

Note: t-statistics are reported in parentheses; *, **, and *** indicate significance levels of 0.10, 0.05, and 0.01, respectively. All specifications include firm and year fixed effects and use heteroskedasticity-robust standard errors.

All estimates of *GWS* in Table 5 are positive and statistically significant, thereby lending further support to the main findings.

### Additional analyses

#### Mechanism analysis.

We examine how greenwashing affects executive compensation in terms of short-term performance and executive reputation. This study employs return on assets (*ROA*) as a short-term performance metric. According to Chen et al. (2025) [53], we measure executive reputation (*Mreputation*) as the natural logarithm of 1 plus the number of positive media reports about executives of listed companies, obtained from the CNRDS database (https://www.cnrds.com/). The results in columns (1) to (2) of Table 3 show that all coefficients are significantly positive at the 1% level, indicating that greenwashing improves short-term firm performance and enhances executive reputation, which in turn increases executive compensation.

#### Heterogeneity tests.

First, we establish a dummy variable (*Soe*) for ownership type, coded as 1 for state-owned enterprises and 0 for non-state-owned enterprises. Column (2) of Table 6 reveals a significantly negative coefficient for the interaction term between ownership and greenwashing at the 1% level. This suggests heterogeneity in our primary findings across different ownership structures, with the promoting effect of greenwashing on executive compensation being predominantly observed in non-state-owned enterprises.

**Table 6:** Additional analyses.

	(1)		(2)	(3)	(4)	
	*Roa*	*Mreputation*	*MPay*	*MPay*	*MPay*	*MPay*
*GWS*	0.001**	0.021*	0.019***	0.006	0.010**	0.001
	(2.574)	(1.695)	(4.009)	(1.334)	(2.544)	(0.309)
Soe			−0.032***			
			(−2.680)			
Soe*GWS			−0.019***			
			(−2.747)			
Equity				0.068***		
				(6.712)		
Equity*GWS				0.013*		
				(1.846)		
Stock					0.034**	
					(2.031)	
Stock*GWS					0.008	
					(0.723)	
Restrict						0.041***
						(2.632)
Restrict*GWS						0.025**
						(2.029)
control	Yes	Yes	Yes	Yes	Yes	Yes
cons	−0.168***	−1.269	9.076***	9.126***	9.095***	9.084***
	(−4.394)	(−1.211)	(26.312)	(26.683)	(26.497)	(26.497)
*N*	12,312	12,312	12,312	12,312	12,312	12,312
adj. *R*^2^	0.614	0.619	0.808	0.808	0.807	0.808
Firm	Yes	Yes	Yes	Yes	Yes	Yes
Year	Yes	Yes	Yes	Yes	Yes	Yes

Note: t-statistics are reported in parentheses; *, **, and *** indicate significance levels of 0.10, 0.05, and 0.01, respectively. All specifications include firm and year fixed effects and use heteroskedasticity-robust standard errors.

Second, we posit that executives with equity-based incentives possess stronger motivation to engage in greenwashing for higher compensation. A dummy variable (*Equity*) indicating the implementation of equity incentive plans is created and incorporated into the baseline regression model, along with an interaction term with greenwashing. The results in Column (3) of Table 6 indicate a significantly positive interaction coefficient between greenwashing and equity incentives. This indicates that the positive effect of greenwashing on executive compensation is primarily observed in firms that implement equity incentive plans.

Furthermore, we examine the heterogeneous effects of equity incentive types. Based on the existing literature, we construct two dummy variables: one for stock options (*Stock*) and another for restricted stock (*Restrict*). As presented in column (4) of Table 6, the interaction term between stock options and greenwashing is positive but statistically insignificant, while the interaction term between restricted stocks and greenwashing is significantly positive at the 10% level. This suggests that the implementation of restricted stocks has a more pronounced enhancing effect on the relationship between greenwashing and executive compensation, whereas the effect of stock options is not significant.

## Discussion and conclusion

### Discussion of findings

This study investigates a seemingly paradoxical question: given the documented risks, why does corporate greenwashing persist? By shifting the focus from corporate-level costs to executive-level benefits, our findings provide a possible explanation. We demonstrate that corporate greenwashing has a significant positive effect on executive compensation. Engaging in symbolic environmental communication serves as an effective strategy for executives to protect and enhance their private economic benefits. This core finding aligns with managerial entrenchment theory.

Our mechanism analysis indicates that greenwashing enhances executive reputation by improving short-term performance. The study not only confirms that executives effectively capture the short-term economic benefits of greenwashing but also introduces reputation capital as a basis for negotiating executive compensation. Furthermore, we find that the effect of greenwashing on executive compensation is more pronounced in non-state-owned enterprises and those with equity incentive plans. It suggests that the flexible, market-oriented compensation schemes in non-state-owned enterprises, while efficient in some respects, may be more susceptible to greenwashing. Conversely, the stricter oversight and socio-political objectives in state-owned enterprises appear to dampen this effect. Additionally, corporations with equity incentives and grant restricted stock are more inclined to inflate company valuations through greenwashing to maximize their stock-based compensation gains. This finding reveals the underlying logic of how modern compensation mechanisms may inadvertently encourage superficial rather than substantive environmental actions, underscoring the urgent need for governance reforms.

To situate these results within the broader literature, our findings converge with studies highlighting the short-term advantages of greenwashing. For instance, Kim and Lyon (2015) [10] argue that greenwashing can generate immediate performance signals, even if at the expense of long-term sustainability, which aligns with our evidence that short-term performance improvements lead to higher executive pay. Similarly, Sun et al. (2024) [12] emphasize how reputation-enhancing behaviors, including symbolic environmental initiatives, strengthen executives’ bargaining power in compensation negotiations, corroborating our reputation mechanism. Partial support also comes from Yang et al. (2020) [11], who note that greenwashing can yield economic benefits, such as low-cost financing, which we extend to executive-level gains. However, unlike the mainstream literature that emphasizes the negative impacts of greenwashing, this paper’s research focuses on personal benefits rather than corporate-level impacts. This difference may stem from varying analytical perspectives: existing studies often adopt a long-term, firm-centric viewpoint, while our study focuses on short-term, executive-interest-oriented incentive mechanisms.

### Theoretical and practical implications

Our study offers several important implications. Theoretically, it makes a significant contribution to the literature on the economic consequences of greenwashing by revealing a direct personal benefit for the key decision-makers, thereby providing a more granular motivational explanation for its prevalence. It also advances executive compensation research by identifying greenwashing as a previously overlooked, yet strategically important, antecedent.

From a practical perspective, current executive appraisal systems, by overly relying on short-term metrics and neglecting the substantive nature of environmental achievements, may inadvertently reward hypocrisy. Therefore, well-designed remuneration packages that ensure environmental disclosures are included and verified are paramount. For regulators, our study highlights the need to mandate and standardize the disclosure of environmental information effectively, as well as to strengthen industry self-regulation to reduce informational asymmetry and make greenwashing more challenging to execute and sustain.

### Limitations and future research

Despite its contributions, this study is subject to several limitations that offer avenues for future research. First, to obtain an exact measurement of greenwashing remains a complex challenge. Although our metrics can effectively identify the extent of greenwashing by companies, they cannot precisely distinguish whether such discrepancies stem from positive (excessive “window dressing”) or negative (underreporting of actions). Future research may continue to expand the range of variables to reveal underlying motivations more precisely. Second, we acknowledge that our findings are primarily grounded in the specific context of China. Therefore, the generalizability of our results to other countries with distinct institutional and cultural settings should be tested. Future cross-national comparative research would be highly valuable. Finally, future research could investigate other contingent factors, such as the role of specific board characteristics (e.g., the presence of a sustainability committee) or media scrutiny, in mitigating or amplifying the relationship we uncovered.

This study examines the consequences of corporate greenwashing and finds that it positively affects executive compensation levels, particularly among companies with equity incentives and those that are not state-owned. Promoting short-term performance and executive reputation are the mechanisms through which greenwashing increases executive compensation. We use these findings as a basis for recommending that compensation packages be rationalized to include environmental disclosure in appraisals. Lastly, the government should effectively regulate the disclosure of environmental information and strengthen industry self-regulation.
